# ^64^Cu-DOTA-Anti-CTLA-4 mAb Enabled PET Visualization of CTLA-4 on the T-Cell Infiltrating Tumor Tissues

**DOI:** 10.1371/journal.pone.0109866

**Published:** 2014-11-03

**Authors:** Kei Higashikawa, Katsuharu Yagi, Keiko Watanabe, Shinichiro Kamino, Masashi Ueda, Makoto Hiromura, Shuichi Enomoto

**Affiliations:** 1 Graduate School of Medicine, Dentistry, and Pharmaceutical Sciences, Okayama University, Okayama, Japan; 2 Japan Society for the Promotion of Science, Tokyo, Japan; 3 Next-generation Imaging Team, RIKEN Center for Life Science Technologies, Kobe, Japan; Technische Universitaet Muenchen, Germany

## Abstract

Cytotoxic T lymphocyte-associated antigen-4 (CTLA-4) targeted therapy by anti-CTLA-4 monoclonal antibody (mAb) is highly effective in cancer patients. However, it is extremely expensive and potentially produces autoimmune-related adverse effects. Therefore, the development of a method to evaluate CTLA-4 expression prior to CTLA-4-targeted therapy is expected to open doors to evidence-based and cost-efficient medical care and to avoid adverse effects brought about by ineffective therapy. In this study, we aimed to develop a molecular imaging probe for CTLA-4 visualization in tumor. First, we examined CTLA-4 expression in normal colon tissues, cultured CT26 cells, and CT26 tumor tissues from tumor-bearing BALB/c mice and BALB/c nude mice by reverse transcription polymerase chain reaction (RT-PCR) analysis and confirmed whether CTLA-4 is strongly expressed in CT26 tumor tissues. Second, we newly synthesized ^64^Cu-1,4,7,10-tetraazacyclododecane-*N*,*N′*,*N″*,*N*‴-tetraacetic acid-anti-mouse CTLA-4 mAb (^64^Cu-DOTA-anti-CTLA-4 mAb) and evaluated its usefulness in positron emission tomography (PET) and ex-vivo biodistribution analysis in CT26-bearing BALB/c mice. High CTLA-4 expression was confirmed in the CT26 tumor tissues of tumor-bearing BALB/c mice. However, CTLA-4 expression was extremely low in the cultured CT26 cells and the CT26 tumor tissues of tumor-bearing BALB/c nude mice. The results suggested that T cells were responsible for the high CTLA-4 expression. Furthermore, ^64^Cu-DOTA-anti-CTLA-4 mAb displayed significantly high accumulation in the CT26 tumor, thereby realizing non-invasive CTLA-4 visualization in the tumor. Together, the results indicate that ^64^Cu-DOTA-anti-CTLA-4 mAb would be useful for the evaluation of CTLA-4 expression in tumor.

## Introduction

Cancer is a complex mixture of host and tumor cells. Whereas the human body has the ability to produce an anti-tumor immune response, cancers develop multiple strategies to evade the host immune system [1]. Cytotoxic T lymphocyte-associated antigen-4 (CTLA-4), also known as cluster of differentiation 152 (CD152), is one of the most important molecules that are involved in the downregulation of the immune system and the anti-tumor response. CTLA-4 is expressed predominantly on the surface of two major subsets of CD4^+^ T cells: regulatory T cells (Tregs) and activated CD4^+^ effector cells, and activated CD8^+^ effector T cells [2], [3]. In addition, recent research showed that various tumor cells also express CTLA-4 [4].

CTLA-4 targeted therapy augments endogenous response to tumor cells, thereby leading to tumor cell death when utilized on its own or with other therapeutic interventions [3]. It is for this reason that CTLA-4 has attracted attention as a target molecule of cancer immunotherapy [5]. Fully human anti-CTLA-4 monoclonal antibodies (mAbs), ipilimumab and tremelimumab, were developed for the treatment of cancer patients. Ipilimumab is the first drug to demonstrate survival benefits in metastatic melanoma patients, and was approved by the US Food and Drug Administration (FDA) for the treatment of advanced melanoma in 2011. Pre-clinical and clinical trials of anti-CTLA-4 mAbs have been conducted for the treatment of other cancers, including colon, breast, lung, ovarian, and prostate cancers [3], [6].

Although CTLA-4-targeted therapy is an attractive method for the treatment of various cancers, the therapy is beset by several problems. First, the enhanced T cell response by the CTLA-4 blockade frequently produces autoimmune-related adverse effects, such as rash, diarrhea, colitis, hepatitis, and hypophysitis [7], [8]. A superagonist antibody for CD28 (TGN1412), which directly stimulates T cells, caused life-threatening inflammatory reactions in a London clinical trial [9]. Extreme precaution must be taken when CTLA-4-targeted antibodies are used for the treatment because CTLA-4 is an antagonist of CD28–ligand interactions [10]. Second, antibody drugs are extremely expensive. One treatment course of ipilimumab in the United States consists of four doses at US$30,000 per dose [2], [11]. Clearly, there is an urgent need to develop a method to screen patients for sensitivity to the CTLA-4-targeted therapy, to eliminate adverse effects brought about by ineffective therapy and reduce unnecessary financial burden in non-sensitive patients. The identification of CTLA-4 expression in tumor prior to molecular targeted therapy would lead to evidence-based and cost-efficient medical care.

Biopsy is principally conducted to evaluate the expression of molecules of interest. However, it is an invasive and stressful procedure. Moreover, biopsy evaluates the expression of target molecules only in a localized region of the tumor. Thus, it is difficult to acquire information of a patient's sensitivity to a molecular targeted drug for tumors existing in whole body.

Molecular imaging can provide molecular information of the whole body in a noninvasive manner and be used for the determination of sensitivity to antibody drugs. Tumor imaging probes for human epidermal growth factor receptor 2 (HER2) [12]–[14], epidermal growth factor receptor (EGFR) [15]–[18], and vascular endothelial growth factor (VEGF) [19], [20], which are the target molecules of trastuzumab, cetuximab/panitumumab, and bevacizumab, respectively, have been developed. The expression of those molecules in tumor was detected with their respective probes by positron emission tomography (PET) or single photon emission computed tomography (SPECT). However, to our knowledge, a molecular imaging probe that targets CTLA-4 has yet to be developed.

In this study, we aimed to develop a molecular imaging probe for CTLA-4 visualization in tumor. First, CTLA-4 expression was examined in CT26 tumor tissues and cultured CT26 cells by reverse transcription polymerase chain reaction (RT-PCR) analysis. Second, we newly developed ^64^Cu-1,4,7,10-tetraazacyclododecane-*N*,*N′*,*N″*,*N‴*-tetraacetic acid (DOTA)-anti-mouse CTLA-4 mAb by introducing DOTA groups to anti-mouse CTLA-4 mAb and subsequent radiolabeling with ^64^Cu. The utility of ^64^Cu-DOTA-anti-CTLA-4 mAb as an imaging probe was assessed by PET imaging and ex-vivo biodistribution analysis. We prepared tumor-bearing mice by syngeneic implantation of CT26 cells (mouse colon tumor cell line) to BALB/c mice for PET imaging. Immune-deprived mice bearing human tumor cell lines were not used because T cells might be responsible for the CTLA-4 expression in the tumor tissues.

## Materials and Methods

### Cell culture

CT26 was purchased from American Type Culture Collection and cultured in RPMI 1640 medium supplemented with 10% fetal bovine serum, 4 mM L-glutamine, 10 U/mL penicillin, and 10 mg/mL streptomycin at 37°C in a humidified atmosphere containing 5% CO_2_.

### Preparation of subcutaneous tumor model mice

Female BALB/c and BALB/c (nu/nu) nude mice (4–6 weeks old) were purchased from CLEA Japan Inc. Tumor-bearing BALB/c and BALB/c nude mice were prepared by subcutaneously implanting CT26 cells (1–4×10^6^ cells). Investigations were initiated after receiving approval from the committee on animal experiments of Okayama University.

### RT-PCR analysis

RNA extraction and cDNA synthesis were conducted by using the same methods as our previous report [21]. Total RNA was isolated from cultured cells and tissues with TRIZOL reagent (Life Technologies Co., Ltd.) and a PureLink RNA Mini Kit (Life Technologies Co., Ltd.). One microgram of total RNA was used as the template for single-strand cDNA synthesis with a Transcriptor First Strand cDNA Synthesis Kit (Roche Co., Ltd.). Analysis of mRNA expression levels was carried out with RT-PCR using TaKaRa Ex Taq (TaKaRa Co., Ltd.). The amplification of β-actin is shown as internal control. Primer sequences are listed in Table S1. The amplicons were separated on agarose gel (AGAROSE I, Amresco, Inc.), stained with ethidium bromide, and visualized with a Benchtop 2UV Transilluminator (UVP, Inc.).

### ^64^Cu-DOTA-anti-CTLA-4 mAb production

Anti-mouse CTLA-4 mAb (200–500 µg) (R&D Systems, Inc.) was conjugated to DOTA-mono-*N*-hydroxysuccinimide ester (DOTA-mono-NHS ester; Macrocyclics, Inc.) in phosphate-buffered saline without calcium and magnesium (pH 7.5) (PBS (−)), by using a 100-fold molar excess of DOTA-mono-NHS ester. The mixture was stirred at room temperature (RT) for three hours to give the DOTA-anti-CTLA-4 antibody. The DOTA-anti-CTLA-4 antibody was purified with a PD-10 column (GE Healthcare Co., Ltd.) and an Amicon-Ultra 50 K device (Millipore Co., Ltd.). The DOTA-anti-CTLA-4 antibody was analyzed by size-exclusion high-performance liquid chromatography (SE-HPLC) using TSK-GEL Super SW3000 (Tosoh Co., Ltd.). The mobile phase of 10 mM PBS (−) containing 0.3 M NaCl was used and the flow rate was 0.35 mL/min.

^64^Cu was produced by irradiating a 99.6% ^64^Ni-enriched nickel target with 12 MeV protons using a cyclotron (CYPRIS-HM12, Sumitomo Heavy Industries, Ltd.). Then, ^64^Cu was purified with a Muromac column (Muromachi Technos Co., Ltd.). The buffer solution of DOTA-anti-CTLA-4 mAb was replaced with 0.1 M acetate buffer (pH 6.5) three times by using an Amicon-Ultra 50 K device (Millipore Co., Ltd.). DOTA-anti-CTLA-4 mAb was radiolabeled with ^64^Cu by incubating at 40°C for one hour. To remove excess ^64^Cu, the buffer was replaced with 0.2 M glycine buffer by using the Amicon-Ultra 50 K device. Buffer of the purified antibody solution was replaced with PBS (−) by using the Amicon-Ultra 50 K device. The resultant solution was used for injection.

The radiochemical purity of ^64^Cu-DOTA-antibodies in PBS (−) was confirmed by reversed phase radio-thin layer chromatography (TLC). This analysis was performed with a TLC aluminum sheet, RP-18 F254 S (Merck Chemicals Co., Ltd.) and methanol∶water∶acetic acid (4∶1∶1) was used as the mobile phase. TLC chromatograms were obtained by autoradiography (FLA-7000IR; GE Healthcare Co., Ltd.). ^64^Cu-DOTA-isotype IgG_2A_ (^64^Cu-DOTA-Control IgG) was produced in the same way as that for negative control by using rat IgG_2A_ isotype control (R&D Systems, Inc.).

### Assay for CTLA-4 binding activity

The CTLA-4 binding activity of DOTA-anti-CTLA-4 mAb and DOTA-Control IgG was examined by enzyme-linked immunosorbent assay (ELISA) and compared with that of original anti-CTLA-4 mAb and DOTA-Control IgG. Twenty ng of recombinant mouse CTLA-4 (R&D Systems, Inc.) in 50 mM carbonate buffer (pH 9.6) per well was added into a 96-well ELISA plate (R&D Systems, Inc.). After blocking with 3% bovine serum albumin (BSA) and 1% Tween 20 in PBS (−) containing 0.05% Tween 20, 5 ng of the antibodies in PBS (−) containing 1% BSA and 0.05% Tween 20 was added to each well and incubated for one hour. After incubation, each well was treated with 50 uL of HRP-conjugated anti-rat IgG (R&D Systems, Inc.) diluted 1∶6000 with PBS (−) containing 1% BSA and 0.05% Tween 20. Peroxidase activity was visualized with a TMB Microwell Peroxidase Substrate System (Kirkegaard & Perry Laboratories, Inc.) and the absorbance at 450 nm was measured. The absorbance was corrected by performing a blank trial. The corrected absorbance values of DOTA-anti-CTLA-4 mAb and DOTA-Control IgG were respectively divided by the absorbance of anti-CTLA-4 mAb, and relative immunoreactivities were calculated.

### Matrix-assisted laser desorption-ionization time-of-flight mass spectrometry (MALDI-TOF-MS) analysis

MALDI-TOF-MS was conducted to determine the extent of DOTA conjugation to antibodies using a method similar to that reported by Lu et al. [22]. MALDI-TOF-MS was performed by using an Ultraflex III MALDI TOF/TOF (Bruker Daltonics Co., Ltd.). Non- and DOTA-conjugated antibodies were desalted with PD Spin Trap G-25 (GE Healthcare Co., Ltd.). Sinapinic acid (Nacalai Tesque, Inc.) at 20 mg/mL in 2∶1 acetonitrile/H_2_O with 0.1% trifluoroacetic acid (Wako Pure Chemical Industries, Co., Ltd.) was used as the MALDI matrix.

### PET imaging study

^64^Cu-DOTA-anti-CTLA-4 mAb (4 µg, approximately 16 MBq) or ^64^Cu-DOTA-Control IgG (4 µg, approximately 14 MBq) was intravenously administered to CT26-bearing BALB/c mice via the tail vein. Forty-eight hours after administration of the radiolabeled antibodies, probe uptake in the CT26-bearing mice was measured with a small-animal PET scanner (microPET Focus220; Siemens Medical Solutions Inc.). During PET imaging, the mice were anesthetized with 1.5% isoflurane and 1.5% N_2_O gas, and placed in the prone position. Emission data were acquired for 60 min. The acquired data were summed into sinograms and three-dimensional images were reconstructed by maximum a posteriori (MAP). Coronal and sagittal images were displayed in 918×760 and 550×760 pixel formats, respectively, with a pixel size of 0.053 mm×0.053 mm. The image intensity was expressed by standardized uptake value (SUV). SUV_max_ was calculated by ASIPRO software package (Concorde Microsystems, Inc.).

### Biodistribution study

Forty-eight hours after administration of ^64^Cu-DOTA-anti-CTLA-4 mAb (4 µg, 1 MBq) or ^64^Cu-DOTA-Control IgG (4 µg, 1 MBq), the animals were immediately sacrificed and the organs and blood were removed. The organs and blood were weighed and radioactivities were counted with a gamma counter (ARC-7001B, ALOKA Co., Ltd.). Decay-corrected uptake was expressed as the percentage of injected dose per gram and calculated as the ratio to blood or muscle for comparison of the accumulation abilities in the CT26 tumor between ^64^Cu-DOTA-anti-CTLA-4 antibody and ^64^Cu-DOTA-Control IgG.

### Immunohistological staining

Tumor-bearing BALB/c mice were sacrificed and CT26 tumor tissues including the normal tissues around them were resected and embedded in Optimal Cutting Temperature (O.C.T.) compound (Sakura Finetek Japan Co., Ltd.). Ten-µm-thick frozen tissue sections were prepared and mounted on MAS-coated glass slides (Matsunami Glass Ind., Co., Ltd.). The tissue sections were fixed with 4% paraformaldehyde in PBS (−), blocked with 5% goat serum in PBS (−), and incubated with anti-CTLA-4 antibody (R&D Systems, Inc.). Then, the tissue sections were subjected to endogenous peroxidase inactivation with 0.19% H_2_O_2_/methanol (Wako Pure Chemical Industries, Ltd.), followed by incubation with horseradish peroxidase conjugated anti-rat IgG antibody (R&D Systems, Inc.). Immunocomplexes were visualized with a DAB substrate kit (Dako Co., Ltd.).

### Statistical analysis

SUV_max_ data are expressed as means ± standard deviation (SD) and other data are expressed as means ± standard error of mean (SEM). Statistical significance was determined using the Student's t-test. The level of significance was taken as p<0.01. The tests were performed using GraphPad Prism software (GraphPad Software, Inc.).

## Results

### CTLA-4 was strongly expressed in CT26 tumor tissues but not cultured CT26 cells

**Figure 1:**
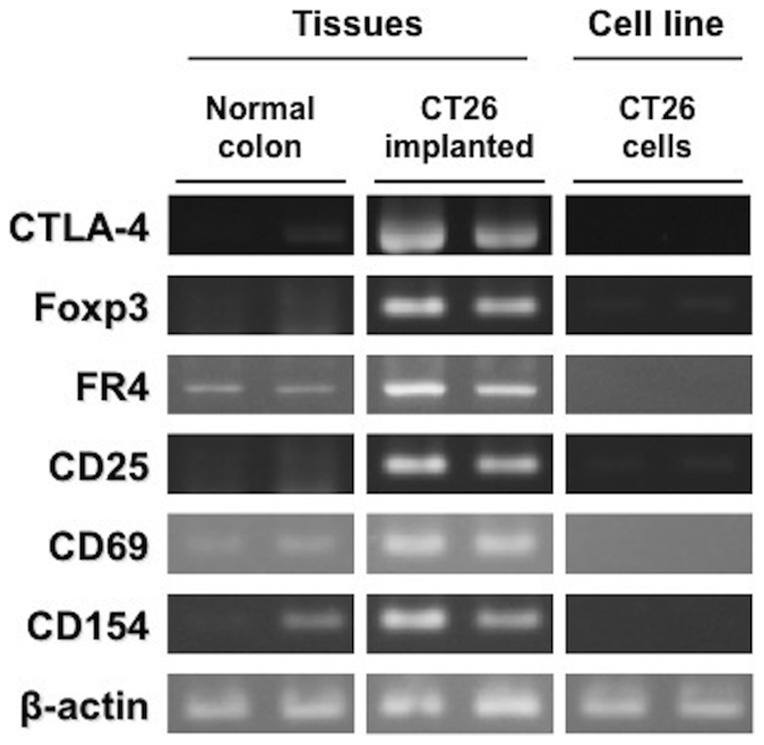
RT-PCR in normal colon tissues, CT26 tumor tissues, and cultured CT26 cells. Expression of CTLA-4, Treg markers, and T cell activation markers in normal colon tissues, CT26 tumor tissues, and cultured CT26 cells.

First, RT-PCR was carried out to examine CTLA-4 expression in CT26 tumor tissues and cultured CT26 cells, and the results were compared to those obtained with normal colon tissues (Fig. 1). CTLA-4 (amplicon length: 920 base pairs (bp)) was strongly expressed in CT26 tumor tissues compared with normal colon tissues.

On the other hand, CTLA-4 expression was extremely low in cultured CT26 cells. Moreover, Treg markers, such as forkhead box P3 (Foxp3) and folate receptor 4 (FR4) [23], [24], were more strongly expressed in the tumor tissues than the normal colon tissues and the cultured CT26 cells. The expression of CD25 and CD69, which are molecules expressed on regulatory and activated T cells [23]–[25], was also increased in the tumor tissues compared to the normal colon tissues and the cultured CT26 cells. CD154, which is induced on T cells by T cell activation [26], was also more strongly expressed in the tumor tissues than the normal colon tissues and the cultured CT26 cells.

### CTLA-4 and T cell marker expression was low in CT26 tumor tissues from tumor-bearing BALB/c nude mice

**Figure 2:**
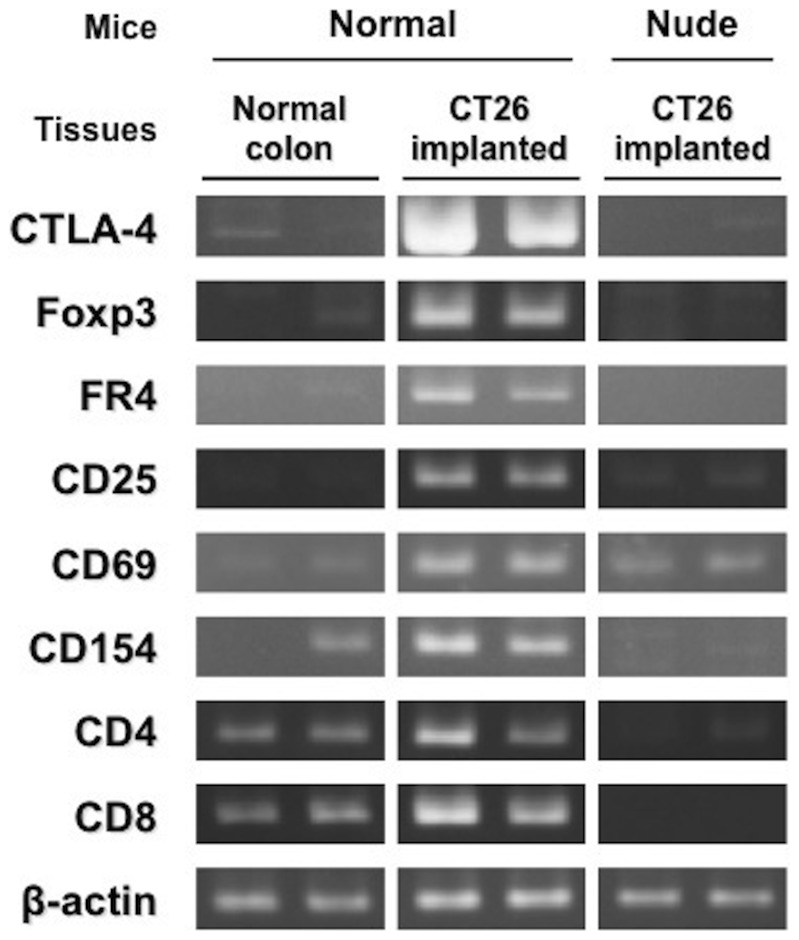
Gene expression analyses in tissues from tumor-bearing BALB/c and BALB/c nude mice. CTLA-4 and T cell marker expression in normal colon tissues from normal BALB/c mice, CT26 tumor tissues from tumor-bearing BALB/c mice, and CT26 tumor tissues from tumor-bearing BALB/c nude mice.

From the results of Fig. 1, we assumed that T cells were involved in CTLA-4 expression in the CT26 tumor tissues from the tumor-bearing BALB/c mice, and CT26 tumor tissues from the tumor-bearing BALB/c nude mice did not express CTLA-4 due to a marked decrease of T cells in those mice. Thus, we prepared two subcutaneous tumor models by syngeneic subcutaneous transplantation of CT26 into normal BALB/c mice or BALB/c nude mice, and compared CTLA-4 and T cell marker expression in the CT26 tumor tissues from the tumor-bearing BALB/c mice with those from the tumor-bearing BALB/c nude mice by RT-PCR analysis (Fig. 2).

RT-PCR showed that CTLA-4 expression was dramatically decreased in the CT26 tumor tissues from the tumor-bearing BALB/c nude mice, compared with those from the tumor-bearing BALB/c mice. Furthermore, we confirmed that the expression of CD4 and CD8 as well as Foxp3, FR4, CD69, CD154, and CD25 was markedly decreased in the tumor tissues from the tumor-bearing BALB/c nude mice.

### DOTA-conjugated antibody probe was synthesized

**Figure 3:**
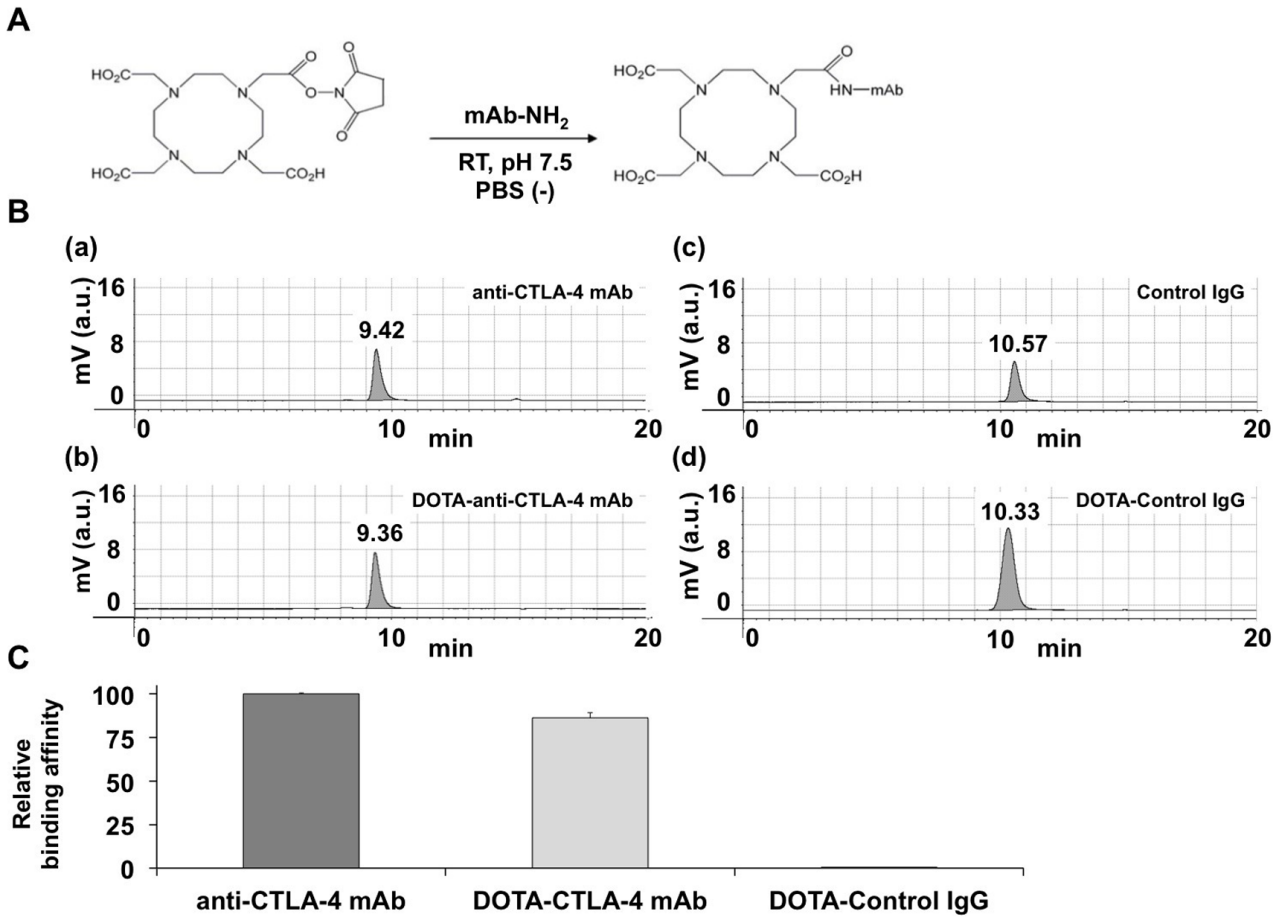
Preparation of DOTA-conjugated mAb. A. Scheme of the synthesis of DOTA-conjugated mAb. B. HPLC analysis of original and DOTA-conjugated mAbs. C. Evaluation of CTLA-4 binding activity of DOTA-anti-CTLA-4 mAb. Data are expressed as means ± SEM.

DOTA chelators were conjugated to each mAb, as shown in Figure 3A. The chromatograms of all the mAbs showed a single peak. In addition, the retention times of anti-CTLA-4 mAb, DOTA-anti-CTLA-4 mAb, Control IgG, and DOTA-Control IgG were 9.42, 9.36, 10.57, and 10.33, respectively (Fig. 3B). The retention times of DOTA-conjugated antibodies were slightly shorter than those of the original antibodies, suggesting that anti-CTLA-4 mAb or Control IgG conjugated to DOTA and was well purified. Furthermore, MALDI-TOF-MS analysis was carried out to measure the average number of DOTA chelators that were conjugated to anti-CTLA-4 mAb or Control IgG (Table 1). The mass differences between anti-CTLA-4 mAb and DOTA-anti-CTLA-4 mAb, and between Control IgG and DOTA-Control IgG were 1634 and 1686, respectively. The mass differences were divided by the mass value of single DOTA conjugation (386 mass units), and the resulting values represented the average number of DOTA chelators that were conjugated to anti-CTLA-4 mAb or Control IgG. From the calculations, 4.2 or 4.4 DOTA chelators on average were conjugated into a single molecule of anti-CTLA-4 mAb or Control IgG.

**Table 1:** Average molecular weights of original and DOTA-conjugated antibodies, and estimated numbers of DOTA chelators per unit antibody.

Antibody	Average molecular weight	Mass difference	The number of DOTA per antibody
anti-CTLA-4 mAb	150097	1634	4.2
DOTA-anti-CTLA-4 mAb	151731		
Control IgG	147870	1686	4.4
DOTA-Control IgG	149557		

Then, the binding activity of DOTA-anti-CTLA-4 mAb to CTLA-4 was measured by ELISA (Figure 3C). The binding activity of DOTA-anti-CTLA-4 mAb to CTLA-4 was 86.3±2.8% of that of the original anti-CTLA-4 mAb. The binding activity of DOTA-Control IgG was 0.3±0.1%.

### ^64^Cu-DOTA-anti-CTLA-4 mAb enabled clear visualization of CTLA-4-positive tumor by PET

**Figure 4:**
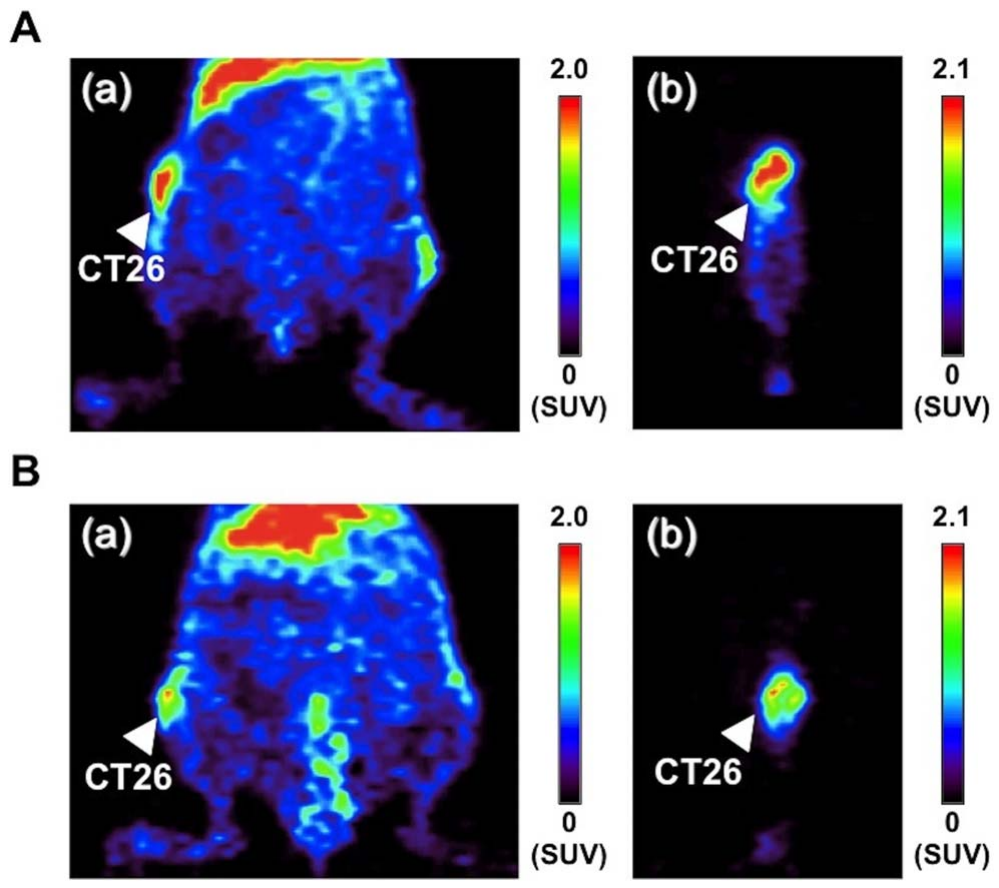
PET images of ^64^Cu-DOTA-anti-CTLA-4 mAb and ^64^Cu-DOTA-Control IgG. A. Representative coronal (a) and sagittal (b) PET images of ^64^Cu-DOTA-anti-CTLA-4 mAb in CT26-bearing mice. B. Representative coronal (a) and sagittal (b) PET images of ^64^Cu-DOTA-Control IgG in CT26-bearing mice.

^64^Cu-DOTA-anti-CTLA-4 mAb and ^64^Cu-DOTA-Control IgG were obtained in radiochemical yields of 94% and 97%, respectively. The radiochemical purities of both probes were higher than 94%. To evaluate the ^64^Cu-DOTA-anti-CTLA-4 mAb uptake by CTLA-4 positive tumor (CT26), we performed PET and ex-vivo biodistribution analysis. Representative coronal and sagittal images are shown in Figure 4. At 48 hours after administration of the probes, ^64^Cu-DOTA-anti-CTLA-4 mAb clearly visualized the CT26 tumors and ^64^Cu-DOTA-anti-CTLA-4 mAb showed higher accumulation in the tumors than ^64^Cu-DOTA-Control IgG (^64^Cu-DOTA-anti-CTLA-4 mAb: SUVmax = 2.65±0.01, n = 2; ^64^Cu-DOTA-Control IgG: SUVmax = 2.06±0.32, n = 2).

**Figure 5:**
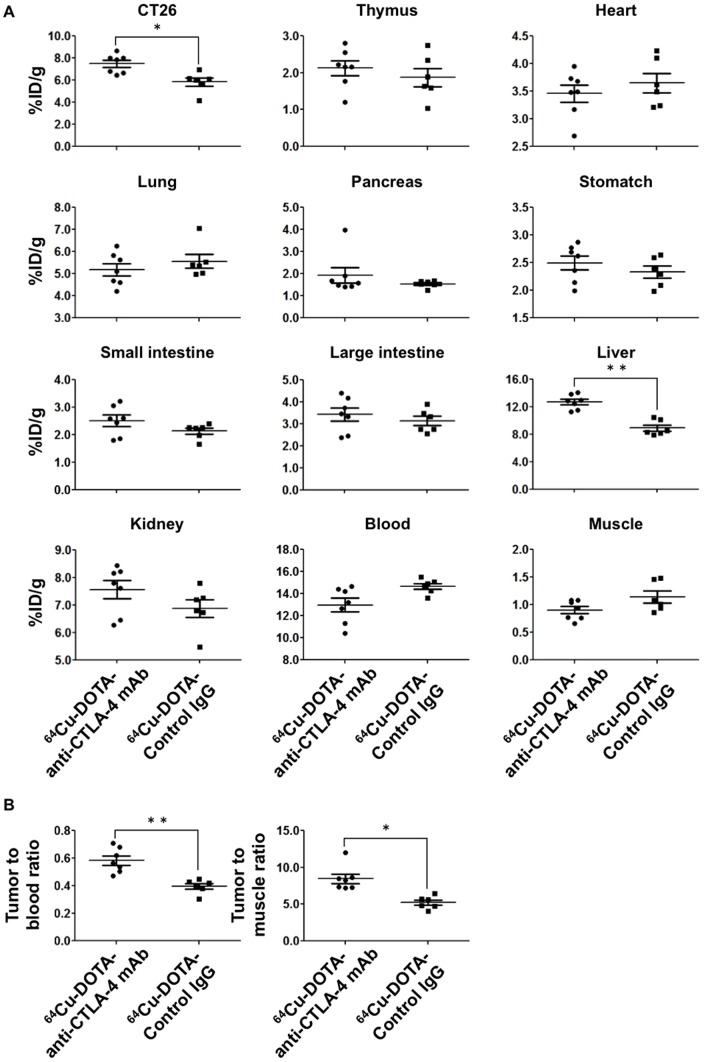
Biodistribution analysis of ^64^Cu-labeled antibody probes. A. %ID/g of ^64^Cu-DOTA-anti-CTLA-4 mAb (n = 7) and ^64^Cu-DOTA-Control IgG (n = 6). B. Tumor-to-blood and tumor-to-muscle ratios of ^64^Cu-DOTA-anti-CTLA-4 mAb (n = 7) and ^64^Cu-DOTA-Control IgG (n = 6). Data are expressed as means ± SEM. Symbols^*^ and ^**^ denote p<0.01 and p<0.001 vs. ^64^Cu-DOTA-Control IgG, respectively.

The results were consistent with those of the ex-vivo biodistribution study (Fig. 5). ^64^Cu-DOTA-anti-CTLA-4 mAb showed significantly higher accumulation in the CT26 tumors than ^64^Cu-DOTA-Control IgG (7.49±0.32%ID/g vs. 5.84±0.38%ID/g, p<0.01). Moreover, ^64^Cu-DOTA-anti-CTLA-4 mAb showed higher tumor-to-blood and tumor-to-muscle ratios than ^64^Cu-DOTA-Control IgG (tumor-to-blood ratio: 0.58±0.03 vs. 0.40±0.02, p<0.001; tumor-to-muscle ratio: 8.48±0.63 and 5.31±0.35, p<0.01).

In addition, CTLA-4 protein expression in the CT26 tumor was confirmed by immunohistochemical staining (Fig. S1A). CTLA-4 was weakly expressed in the normal tissues surrounding the tumor (Fig. S1B).

## Discussion

CT26 is a N-nitroso-N-methylurethane-induced, undifferentiated colon carcinoma cell line and recent cancer immunotherapy studies have shown that CTLA-4 blockade reduced CT26 colon tumor size and was effective in CT26 tumor models [27], [28]. Therefore, in this study, we used the CT26 cell line to prepare subcutaneous tumor models for PET imaging.

First, we compared CTLA-4 expression in CT26 tumor tissues, normal colon tissues, and/or cultured CT26 cells by RT-PCR analyses and confirmed that CTLA-4 was strongly expressed in the CT26 tumor tissues compared to the normal colon tissues. There are four functionally different forms of CTLA-4: the full-length form (containing exons 1–4), the soluble form (exons 1, 2, and 4), the ligand-independent form (exons 1, 3, and 4), and the form containing only exons 1 and 4 [29]–[34]. In our experiments, the full-length form of CTLA-4 (amplicon length: 920 bp), which is a representative immunosuppressive form of CTLA-4, was expressed in the CT26 tumor tissues.

On the other hand, we found that CTLA-4 was not expressed in the cultured CT26 cells, although it was strongly expressed in the CT26 tumor tissues. Contardi et al. reported some human tumor cell lines that expressed CTLA-4 [4]. On the other hand, CTLA-4 is expressed also on CD25^+^ (and/or) Foxp3^+^ (and/or) FR4^+^ CD4^+^ Tregs, activated CD4^+^ effector T cells, and activated CD8^+^ effector T cells [2], [3], [24]. In addition, flow cytometry analysis by Valzasina et al. revealed that almost all CD4^+^ T cells in the CT26 tumor tissues expressed CD25 [35]. From those reports, we hypothesized that CTLA-4 expression in the CT26 tumor tissues regulated by T cells. To prove our hypothesis, we examined the expression of several T cell markers in normal colon tissues, CT26 tumor tissues, and cultured CT26 cells. RT-PCR analysis showed that Treg markers and T cell activation markers were strongly expressed in the CT26 tumor tissues but not the normal colon tissues or cultured CT26 cells. Therefore, we assumed that the T cells were responsible for CTLA-4 expression in the CT26 tumor tissues. Then, we used RT-PCR to compare the expression of CTLA-4 and T cell markers in the CT26 tumor tissues of tumor-bearing BALB/c mice with those of tumor-bearing BALB/c nude mice in hopes of elucidating the relationship between CTLA-4 expression in the CT26 tumor tissues and T cells. BALB/c nude mice are thymus-deficient and thus, the number of T cells is greatly reduced in those mice. Interestingly, we found that the expression of CTLA-4 as well as T cell markers was quite low in the CT26 tumor tissues from the BALB/c nude mice. The results indicated that T cells were responsible for the CTLA-4 expression.

Second, we developed a molecular imaging probe that targets CTLA-4 and examined its utility in mice bearing CTLA-4-expressing CT26 tumor. The anti-CTLA-4 mAb for the imaging probe synthesis was made by using recombinant mouse CTLA­4 representing an extracellular domain of mouse CTLA-4 (Ala36­Phe161) as the immunogen. We selected this mAb for CTLA-4 imaging probe synthesis because clinically used anti-CTLA-4 mAb (ipilimumab) also recognizes the extracellular domain of CTLA-4 and a mAb probe that recognizes the extracellular domain of CTLA-4 is suited for the prediction of the efficacy and drug disposition of ipilimumab.

For CTLA-4 imaging, we conjugated anti-CTLA-4 mAb to DOTA. DOTA-conjugated mAb was prepared by reacting the nucleophilic amino group in the amino acid residue (particularly in lysine) of mAb with the electrophilic DOTA-mono-NHS ester. The binding activity of antibodies to CTLA-4 may be reduced, particularly when DOTA conjugates to lysine residues critical for mAb binding to CTLA-4 [36]. Therefore, it is necessary to check in advance the binding activities of the antibodies. We examined the binding activity of DOTA-conjugated anti-CTLA-4 mAb and compared it with that of control IgG. ELISA confirmed that the binding activity of DOTA-anti-CTLA-4 mAb was preserved for use in CTLA-4 imaging although a slight reduction (86.3±2.8%) was observed relative to the binding activity of the original anti-CTLA-4 mAb. The binding activity of DOTA-Control IgG was extremely low (0.3±0.1%) compared with that of DOTA-anti-CTLA-4 mAb. The results indicate the successful preparation of metal-chelator-conjugated anti-CTLA-4 mAb having CTLA-4 binding activity.

The choice of the positron emitter is an important factor for successful PET imaging. In our study, ^64^Cu was used for labeling mAbs. ^64^Cu decay generates positron emissions applicable to PET and the half-life of ^64^Cu (T_1/2_ = 12.7 h) is sufficiently long for imaging up to 24 to 48 h after administration to accommodate the mAb localization time. Therefore, ^64^Cu has been used for the development of mAb-based radiopharmaceuticals [13], [15], [19]. In our preliminary biodistribution analyses, tumor uptake on ^64^Cu-DOTA-anti-CTLA-4 mAb was not significantly higher than that on ^64^Cu-DOTA-Control IgG at 24 h after administration of the probes (data was not shown). Therefore, we chose later time point (48 h) for PET imaging and ex-vivo biodistribution analysis.

The utility of ^64^Cu-DOTA-anti-CTLA-4 mAb was examined by PET and ex-vivo biodistribution analysis. We were able to visualize CTLA-4-positive tumor by PET with ^64^Cu-DOTA-anti-CTLA-4 mAb as the probe. Ex-vivo biodistribution analysis also revealed that ^64^Cu-DOTA-anti-CTLA-4 mAb showed significant accumulation in the CT26 tumor compared to ^64^Cu-DOTA-Control IgG. The results indicated that ^64^Cu-DOTA-anti-CTLA-4 mAb is useful for the noninvasive imaging of CTLA-4 expression in tumor. Furthermore, we quantified PET images and calculated ^64^Cu-DOTA-anti-CTLA-4 mAb/^64^Cu-DOTA-Control IgG ratio in the CT26. The mean ratio of SUV_max_ values in CT26 was 1.29 in the PET image and that in CT26 was 1.28 in the ex-vivo biodistribution analysis; further, the quantitative value of PET was similar to the ex-vivo biodistribution data. Thus, quantitativity was ensured in our PET experiment. In addition, although further investigation is needed, ^64^Cu-DOTA-anti-CTLA-4 mAb could be used in the diagnosis of other types of tumor invaded by T cells, regardless of CTLA-4 expression in the tumor cells.

In conclusion, we have developed ^64^Cu-DOTA-anti-CTLA-4 mAb and evaluated its potential as a new radiotracer for the noninvasive evaluation of CTLA-4 expression in tumor. Our results demonstrated that ^64^Cu-DOTA-anti-CTLA-4 mAb visualized CTLA-4 expression in CT26 tumor in a noninvasive manner. Therefore, ^64^Cu-DOTA-anti-CTLA-4 mAb is useful for evaluating CTLA-4 expression in the tumor. The evaluation of CTLA-4 expression in tumors using ^64^Cu-DOTA-anti-CTLA-4 mAb would enable selection of patients sensitive to CTLA-4-targeted therapy, thereby eliminating the adverse effects brought about by ineffective therapy and reducing unnecessary financial burden in non-sensitive patients.

## Supporting Information

Figure S1 **Immunohistochemically stained images of CTLA-4 in representative CT26 tumor and normal tissue sections.** A. CT26 tumor tissue section. B. Normal tissue section surrounding CT26 tumor tissue. Scale bar = 50 ?m.
(DOC)

Table S1 **List of primer sequences for RT-PCR.**
(DOC)

## References

[1] Yaguchi T, Sumimoto H, Kudo-Saito C, Tsukamoto N, Ueda R, et al (2011) The mechanisms of cancer immunoescape and development of overcoming strategies. Int J Hematol 93: 294–300. 21374075 10.1007/s12185-011-0799-6

[2] Pardoll DM (2012) The blockade of immune checkpoints in cancer immunotherapy. Nat Rev Cancer 12: 252–264. 22437870 10.1038/nrc3239 PMC4856023

[3] Grosso JF, Jure-Kunkel MN (2013) CTLA-4 blockade in tumor models: an overview of preclinical and translational research. Cancer Immun 13: 5. 23390376 PMC3559193

[4] Contardi E, Palmisano GL, Tazzari PL, Martelli AM, Fala F, et al (2005) CTLA-4 is constitutively expressed on tumor cells and can trigger apoptosis upon ligand interaction. Int J Cancer 117: 538–550. 15912538 10.1002/ijc.21155

[5] Lesterhuis WJ, Haanen JBAG, Punt CJA (2011) Cancer immunotherapy - revisited. Nat Rev Drug Discov 10: 591–600. 21804596 10.1038/nrd3500

[6] Ribas A, Hanson DC, Noe DA, Millham R, Guyot DJ, et al (2007) Tremelimumab (CP-675,206), a cytotoxic T lymphocyte-associated antigen 4 blocking monoclonal antibody in clinical development for patients with cancer. Oncologist 12: 873–883. 17673618 10.1634/theoncologist.12-7-873

[7] Torino F, Barnabei A, De Vecchis L, Salvatori R, Corsello SM (2012) Hypophysitis induced by monoclonal antibodies to cytotoxic T lymphocyte antigen 4: challenges from a new cause of a rare disease. Oncologist 17: 525–535. 22477725 10.1634/theoncologist.2011-0404 PMC3336822

[8] Weber JS, Kahler KC, Hauschild A (2012) Management of immune-related adverse events and kinetics of response with ipilimumab. J Clin Oncol 30: 2691–2697. 22614989 10.1200/JCO.2012.41.6750

[9] Suntharalingam G, Perry MR, Ward S, Brett SJ, Castello-Cortes A, et al (2006) Cytokine storm in a phase 1 trial of the anti-CD28 monoclonal antibody TGN1412. N Engl J Med 355: 1018–1028. 16908486 10.1056/NEJMoa063842

[10] Walker LSK, Sansom DM (2011) The emerging role of CTLA4 as a cell-extrinsic regulator of T cell responses. Nat Rev Immunol 11: 852–863. 22116087 10.1038/nri3108

[11] Sondak VK, Smalley KS, Kudchadkar R, Grippon S, Kirkpatrick P (2011) Ipilimumab. Nat Rev Drug Discov 10: 411–412. 21629286 10.1038/nrd3463

[12] Dijkers EC, Oude Munnink TH, Kosterink JG, Brouwers AH, Jager PL, et al (2010) Biodistribution of 89Zr-trastuzumab and PET imaging of HER2-positive lesions in patients with metastatic breast cancer. Clin Pharmacol Ther 87: 586–592. 20357763 10.1038/clpt.2010.12

[13] ClinicalTrials.gov website. Available: http://clinicaltrials.gov/ct2/show/NCT00605397. Accessed 2014 Sep 20.

[14] ClinicalTrials.gov website. Available: http://clinicaltrials.gov/ct2/show/NCT00474578. Accessed 2014 Sep 20.

[15] Cai WB, Chen K, He LN, Cao QH, Koong A, et al (2007) Quantitative PET of EGFR expression in xenograft-bearing mice using Cu-64-labeled cetuximab, a chimeric anti-EGFR monoclonal antibody. Eur J Nucl Med Mol Imaging 34: 850–858. 17262214 10.1007/s00259-006-0361-6

[16] Niu G, Li Z, Xie J, Le QT, Chen X (2009) PET of EGFR antibody distribution in head and neck squamous cell carcinoma models. J Nucl Med 50: 1116–1123. 19525473 10.2967/jnumed.109.061820 PMC6435376

[17] Bhattacharyya S, Kurdziel K, Wei L, Riffle L, Kaur G, et al (2013) Zirconium-89 labeled panitumumab: a potential immuno-PET probe for HER1-expressing carcinomas. Nucl Med Biol 40: 451–457. 23454247 10.1016/j.nucmedbio.2013.01.007 PMC3637856

[18] ClinicalTrials.gov website. Available: http://clinicaltrials.gov/show/NCT00691548. Accessed 2014 Sep 20.

[19] Paudyal B, Paudyal P, Oriuchi N, Hanaoka H, Tominaga H, et al (2011) Positron emission tomography imaging and biodistribution of vascular endothelial growth factor with 64Cu-labeled bevacizumab in colorectal cancer xenografts. Cancer Sci 102: 117–121. 21070475 10.1111/j.1349-7006.2010.01763.x

[20] Nagengast WB, de Korte MA, Oude Munnink TH, Timmer-Bosscha H, den Dunnen WF, et al (2010) 89Zr-bevacizumab PET of early antiangiogenic tumor response to treatment with HSP90 inhibitor NVP-AUY922. J Nucl Med 51: 761–767. 20395337 10.2967/jnumed.109.071043

[21] Higashikawa K, Akada N, Yagi K, Watanabe K, Kamino S, et al (2011) Exploration of target molecules for molecular imaging of inflammatory bowel disease. Biochem Biophys Res Commun 410 (3) 416–21. 21672527 10.1016/j.bbrc.2011.05.146

[22] Lu SX, Takach EJ, Solomon M, Zhu Q, Law SJ, et al (2005) Mass spectral analyses of labile DOTA-NHS and heterogeneity determination of DOTA or DM1 conjugated anti-PSMA antibody for prostate cancer therapy. J Pharm Sci 94: 788–797. 15729708 10.1002/jps.20289

[23] Sakaguchi S, Miyara M, Costantino CM, Hafler DA (2010) FOXP3+ regulatory T cells in the human immune system. Nat Rev Immunol 10: 490–500. 20559327 10.1038/nri2785

[24] Yamaguchi T, Hirota K, Nagahama K, Ohkawa K, Takahashi T, et al (2007) Control of immune responses by antigen-specific regulatory T cells expressing the folate receptor. Immunity 27: 145–159. 17613255 10.1016/j.immuni.2007.04.017

[25] Simms PE, Ellis TM (1996) Utility of flow cytometric detection of CD69 expression as a rapid method for determining poly- and oligoclonal lymphocyte activation. Clin Diagn Lab Immunol 3: 301–304. 8705673 10.1128/cdli.3.3.301-304.1996 PMC170336

[26] Elgueta R, Benson MJ, de Vries VC, Wasiuk A, Guo Y, et al (2009) Molecular mechanism and function of CD40/CD40L engagement in the immune system. Immunol Rev 229: 152–172. 19426221 10.1111/j.1600-065X.2009.00782.x PMC3826168

[27] Selby MJ, Engelhardt JJ, Quigley M, Henning KA, Chen T, et al (2013) Anti-CTLA-4 Antibodies of IgG2a Isotype Enhance Antitumor Activity through Reduction of Intratumoral Regulatory T Cells. Cancer Immunol Res 1: 32–42. 24777248 10.1158/2326-6066.CIR-13-0013

[28] Mitsui J, Nishikawa H, Muraoka D, Wang L, Noguchi T, et al (2010) Two distinct mechanisms of augmented antitumor activity by modulation of immunostimulatory/inhibitory signals. Clin Cancer Res 16: 2781–2791. 20460483 10.1158/1078-0432.CCR-09-3243

[29] Teft WA, Kirchhof MG, Madrenas J (2006) A molecular perspective of CTLA-4 function. Annu Rev Immunol 24: 65–97. 16551244 10.1146/annurev.immunol.24.021605.090535

[30] Ueda H, Howson JM, Esposito L, Heward J, Snook H, et al (2003) Association of the T-cell regulatory gene CTLA4 with susceptibility to autoimmune disease. Nature 423: 506–511. 12724780 10.1038/nature01621

[31] Vijayakrishnan L, Slavik JM, Illes Z, Greenwald RJ, Rainbow D, et al (2004) An autoimmune disease-associated CTLA-4 splice variant lacking the B7 binding domain signals negatively in T cells. Immunity 20: 563–575. 15142525 10.1016/s1074-7613(04)00110-4

[32] Araki M, Chung D, Liu S, Rainbow DB, Chamberlain G, et al (2009) Genetic Evidence That the Differential Expression of the Ligand-Independent Isoform of CTLA-4 Is the Molecular Basis of the Idd5.1 Type 1 Diabetes Region in Nonobese Diabetic Mice. J Immunol 183: 5146–5157. 19783679 10.4049/jimmunol.0802610 PMC2871291

[33] Liu SM, Sutherland APR, Zhang Z, Rainbow DB, Quintana FJ, et al (2012) Overexpression of the CTLA-4 Isoform Lacking Exons 2 and 3 Causes Autoimmunity. J Immunol 188: 155–162. 22124121 10.4049/jimmunol.1102042 PMC3245861

[34] Gerold KD, Zheng PL, Rainbow DB, Zernecke A, Wicker LS, et al (2011) The Soluble CTLA-4 Splice Variant Protects From Type 1 Diabetes and Potentiates Regulatory T-Cell Function. Diabetes 60: 1955–1963. 21602513 10.2337/db11-0130 PMC3121435

[35] Valzasina B, Piconese S, Guiducci C, Colombo MP (2006) Tumor-induced expansion of regulatory T cells by conversion of CD4(+)CD25(−) lymphocytes is thymus and proliferation independent. Cancer Res 66: 4488–4495. 16618776 10.1158/0008-5472.CAN-05-4217

[36] Knowles SM, Wu AM (2012) Advances in immuno-positron emission tomography: antibodies for molecular imaging in oncology. J Clin Oncol 30: 3884–3892. 22987087 10.1200/JCO.2012.42.4887 PMC3478579

