# Supplementary material for: 64Cu-DOTA-Anti-CTLA-4 mAb Enabled PET Visualization of CTLA-4 on the T-Cell Infiltrating Tumor Tissues
Source: PLoS One. 2014 Nov 3;9(11):e109866. doi: 10.1371/journal.pone.0109866 (PMC4217715; doi:10.1371/journal.pone.0109866)
Supplement: Figure S1 — Immunohistochemically stained images of CTLA-4 in representative CT26 tumor and normal tissue sections. A. CT26 tumor tissue section. B. Normal tissue section surrounding CT26 tumor tissue. Scale bar = 50 ?m. (DOC) [file pone.0109866.s001.doc]

**Figure S1**

Immunohistochemically stained images of CTLA-4 in representative CT26 tumor and normal tissue sections.

A. CT26 tumor tissue section. B. Normal tissue section surrounding CT26 tumor tissue. Scale bar = 50 μm.
