# Supplementary material for: 64Cu-DOTA-Anti-CTLA-4 mAb Enabled PET Visualization of CTLA-4 on the T-Cell Infiltrating Tumor Tissues
Source: PLoS One. 2014 Nov 3;9(11):e109866. doi: 10.1371/journal.pone.0109866 (PMC4217715; doi:10.1371/journal.pone.0109866)
Supplement: Table S1 — List of primer sequences for RT-PCR. (DOC) [file pone.0109866.s002.doc]

**Table S1**

List of primer sequences for RT-PCR

| Gene | Primer | Sequence (5' to 3') |
| --- | --- | --- |
| CTLA-4 | Forward | ggttttactctgctccctgaggacc |
|  | Reverse | atcccagctctctgttcatgctcc |
| Foxp3 | Forward | tactcgcatgttcgcctacttc |
|  | Reverse | agggattggagcacttgttg |
| FR4 | Forward | atggcacagtggtggcagat |
|  | Reverse | tcagggatggaacaacaggc |
| CD25 | Forward | ttcccacaacccacagaaac |
|  | Reverse | agccgttaggtgaatgcttg |
| CD154 | Forward | cgttgtaagcgaagccaacag |
|  | Reverse | ctcccgattagagcagaaggtg |
| CD69 | Forward | tctggtgaactggaacattgg |
|  | Reverse | agtggaagtttgcctcacagtc |
| CD49d | Forward | accatcagcttgctacttggac |
|  | Reverse | ccacctttgggtagcttctttc |
| CD4 | Forward | gtttccttgggctctgcatc |
|  | Reverse | cagcgtgtctgctacattcatc |
| CD8 | Forward | ttttctgccatgagggacac |
|  | Reverse | atcacaggcgaagtccaatc |
| β-actin | Forward | tctttgcagctccttcgttg |
|  | Reverse | acccattcccaccatcacac |
